# Adapting to new challenges in medical education: a three-step digitization approach for blended learning

**DOI:** 10.1186/s12909-024-05503-1

**Published:** 2024-05-28

**Authors:** Morris Gellisch, Gabriela Morosan-Puopolo, Beate Brand-Saberi, Thorsten Schäfer

**Affiliations:** 1 Center for Medical Education, Ruhr-University Bochum, 44801 Bochum, Germany; 2 Department of Anatomy and Molecular Embryology, Institute of Anatomy, Medical Faculty, Ruhr University Bochum, 44801 Bochum, Germany

**Keywords:** Blended Learning, Digital Education, Medical Education, Educational Technology, Curriculum Development, Stress and Emotions in Education

## Abstract

This research work focuses on evaluating the development of our three-step digitization approach, designed to transform traditional lectures into engaging digital learning experiences. Aimed at offering an easy-to-use solution for educators, this approach addresses the pressing challenges of modern educational environments by enhancing accessibility, engagement, and effectiveness of learning through digital means.

Quantitative analysis demonstrated a notable increase in knowledge gain for students engaged with the digital format (t(230) = -2.795, *p* = 0.006) over traditional methods. Additionally, it was observed that the online setting significantly improved concentration levels (t(230) = -5.801, *p* < .001) and reduced distractions (t(230) = 2.848, *p* = 0.005). Emotional assessments, based on the Achievement Emotions Questionnaire (AEQ), indicated an elevation in enjoyment (t(230) = -4.717, *p* < .001) and a reduction in anxiety (t(230) = 9.446, *p* < .001) within the digital learning environment. A substantial preference for the digital course format was expressed by 61.0% of participants, with 71.4% acknowledging its superior efficiency compared to 14.3% for traditional lectures.

Qualitative feedback underscored the digital format's flexibility, comprehensive learning experience, and immediate feedback as key benefits. Nevertheless, nuances such as a preferred understanding in face-to-face interactions and the absence of a social component in digital settings were noted.

To conclude, the findings from this study illuminate the significant advantages of the three-step digitization approach in meeting contemporary educational challenges. By facilitating an enhanced knowledge acquisition and fostering a supportive emotional climate, this approach signifies a promising direction for the future of medical education and beyond, fusing the convenience of digital solutions with the depth and engagement of traditional learning methodologies.

**Supplementary Information:**

The online version contains supplementary material available at 10.1186/s12909-024-05503-1.

## Introduction

In anticipation of changes within Germany, a new Medical Licensing Regulation is expected to come into effect on October 1, 2027, following a nationwide agreement. This regulation, stemming from the ‘Masterplan Medizinstudium 2020’ agreed upon in 2017, aims to significantly modernize medical education in Germany. The reforms include a shift towards competency-based education, integrating the National Competence-Based Catalogue of Learning Objectives for Medicine (NKLM) into the curriculum, reducing traditional lectures by 30% in favor of digital blended-learning formats, and fostering guided self-study. These changes underscore a broader shift towards more practical and digital-focused medical education. Additionally, our manuscript addresses the increasing demand for digital learning environments, reflecting a global trend towards integrating technology into education. By showcasing the efficacy and student engagement in digital formats, we contribute to the discourse on digital transformation in medical education, aligning with both national and international educational trends.. This approach, tailored to bridge the gap between traditional and digital education, enables instructors, irrespective of their didactic expertise, to seamlessly transition to digital and blended learning models. This straightforward digitization strategy not only aligns with the upcoming legislative requirements but also simplifies the integration of digital tools into medical education.

The imperative of digital learning in medical education is increasingly recognized, as it adapts to the dynamic demands of healthcare. Haag et al. [1] call for a national "Medical Education in the Digital Age" initiative, emphasizing the need for digital skills and technologies in healthcare education. This reflects a growing consensus on the benefits of digital methodologies in medical training. Digital education's role in enhancing communication skills, a vital aspect of medical training, is notable, with evidence suggesting its efficacy might even surpass traditional methods in some instances [2]. Additionally, the integration of technology in medical education is crucial, especially in fields like psychiatry where telepsychiatry and digital continuing education are becoming increasingly important [3]. Innovative educational models like the hybrid ‘flipped classroom’, which uses online resources for concept learning, are proposed to improve learning efficiency and engagement [4]. The effectiveness of digital education technologies, such as high-fidelity mannequins and virtual reality, has been demonstrated in pediatrics, showing their potential to be as effective as, or even more so than, traditional methods [5].

While digital learning has transformed medical education, it's not without drawbacks. Over-reliance on digital tools may diminish memorization skills and lead to a dependency that could impact the depth of medical knowledge retention [6]. The shift to online learning, accelerated by the COVID-19 pandemic, has highlighted concerns regarding digital eye strain and mental health effects, questioning the sustainability of prolonged digital education [7–9]. The growing dependence on digital resources also necessitates enhanced digital literacy among learners for effective comprehension and navigation [10]. In regions with limited resources, e-learning offers a solution to expand access to medical education, addressing challenges like faculty shortages and infrastructure limitations [11]. A new digital divide underscores disparities in the ability to engage with digital education, necessitating the redesign of learning environments to ensure inclusivity [12]. Despite the advantages of e-learning, its integration into medical curricula requires careful consideration to complement rather than replace essential traditional training elements [13, 14]. Transitioning from traditional to online learning further highlights significant engagement challenges, including difficulties in maintaining student interest and ensuring access to essential technologies. In synchronous online teaching, creating an environment that fosters knowledge growth is notably more challenging than in conventional settings, with engaging teachers and students with digital tools proving difficult [15, 16]. In psychobiological research approaches, an interpretative framework could be developed in which correlations between actual physiological activation and engagement during the respective learning unit could be established: It could be shown that the mere transfer of a course to digital teaching is associated with considerable reductions in students' physiological arousal [17], leading to the recommendation that the development of digital learning environments should be accompanied by the implementation of activating and interactive teaching strategies in order to preserve the feeling of engagement in digital educational scenarios [18].

This necessity aligns with findings on the pivotal role of emotions in the learning process. Emotions significantly influence engagement, self-regulation, and learners' appraisal of their performance and outcomes, demonstrating the complexity and importance of emotional experiences in educational settings [19, 20]. Emotions not only intensify all aspects of human behavior, including learning, but are also fundamental in driving attention, memory, motivation, and problem-solving [21]. Given their significant impact on cognitive processes, understanding the influence of both positive and negative emotions on learning and memory is crucial for developing effective educational strategies [21, 22].

Building on the understanding that emotions, active learning, and engagement play critical roles in educational success, our three-step digitization approach is fundamentally grounded in literature, drawing inspiration from established educational research to enhance digital learning environments. It aims to enhance digital learning by designing activities that boost student engagement and lead to meaningful learning outcomes [23]. Strategies for fostering collaborative virtual classes and addressing remote learning challenges are central to our design, promoting interactive learning experiences as a fundamental component of student engagement [23]. This approach is further supported by a framework that advocates for active student engagement through adjusted teaching pedagogies and the integration of educational technologies in an e-learning management system [24]. Evidence from studies in specialized fields, such as General Biology, demonstrates the effectiveness of hybrid active learning interventions in improving learning gains and student perceptions [25]. Additionally, the successful facilitation of active learning in online environments, as seen in ecology education, underscores the value of incorporating online assignments and active learning strategies to enhance the online learning experience and to minimize lecturing when possible [26]. These insights collectively guide the implementation of our digitization approach, ensuring it is rooted in proven strategies for maximizing student interaction, engagement, and success in online learning environments.

Our evaluation of the three-step digitization approach involved a comparative analysis with its traditional face-to-face counterpart, examining aspects such as knowledge acquisition, learning efficiency, mode preferences, and the advantages and disadvantages of each format. Central to our assessment, however, was the emotional response elicited by the digitized lecture, emphasizing the significant role of emotional engagement in educational success. This focus is supported by the work of Pekrun et al. [19], which elucidates the profound impact of academic emotions on self-regulated learning, motivation, and achievement. Their research highlights the complexity of emotions in academic contexts and their direct influence on students' learning strategies and outcomes. By integrating the evaluation of emotional responses into our study, we align with the growing recognition of the importance of emotional aspects in learning, affirming the relevance of our approach in enhancing educational psychology's understanding of digital learning environments.

## Material and methods

**Fig. 1:**
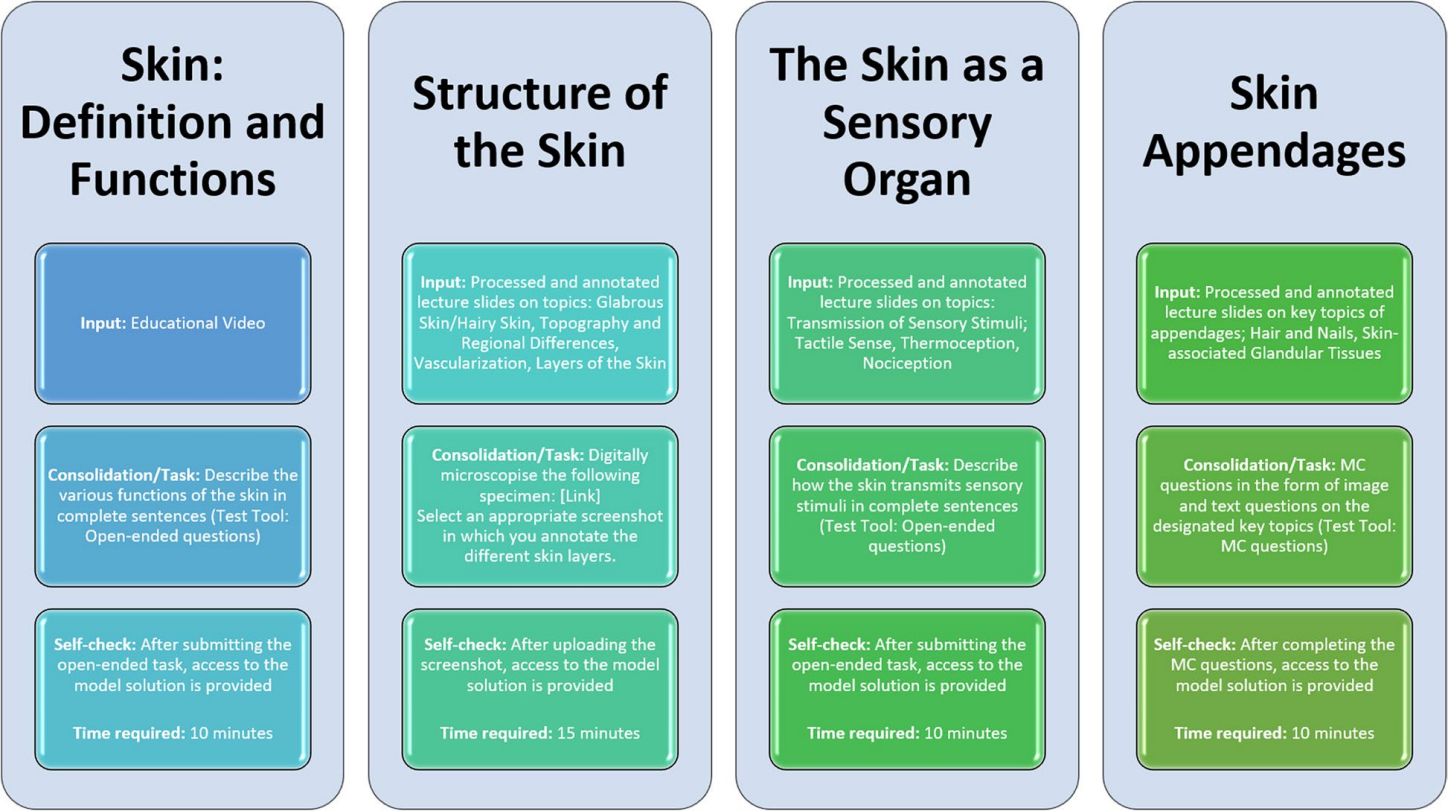
Illustration of the Three-Step Digitization Approach using the example of a teaching unit in Microscopic Anatomy on the topic of skin and skin appendages; this figure depicts the structured process of transforming traditional lecture content into an engaging, interactive digital learning experience. Each thematic focus is divided into three main sections, each representing a sequential step in the digitization process. Step 1—Input: The first section illustrates the initial phase where core topics from the lecture material are presented through digital mediums such as short videos or commented slides. This step is designed to introduce the subject matter and stimulate student interest. Step 2—Consolidation/Task: Following the introductory input, the second section shows the incorporation of active learning tasks. These are practical, application-oriented activities that encourage students to engage deeply with the content, applying what they have learned in simulated scenarios or problem-solving exercises. Step 3—Self-Assessment: The final section outlines the self-assessment phase, where solutions to the active learning tasks are provided. This allows students to independently evaluate their understanding and mastery of the material by comparing their responses with the provided answers, facilitating a reflective learning process

The development of our three-step digitization approach was systematically carried out to transform traditional lecture content into an interactive and engaging digital format. This process started with an in-depth review of lecture materials to pinpoint the main topics and objectives, guiding the creation of digital modules tailored to enhance student engagement and learning efficacy. In the first step, we introduced each topic with brief digital inputs, such as videos or commented slides, aiming to spark initial interest and lay the groundwork for deeper exploration. Following this, the second step engaged students in active learning tasks that encouraged the practical application of the concepts introduced earlier. These tasks were partially designed to simulate real-world scenarios, challenging students to think critically and apply their knowledge. The third step, crucial for reinforcing learning, involved providing students with solutions to the tasks undertaken in step two. This enabled self-assessment, allowing students to independently evaluate their understanding and grasp of the material by comparing their answers with the provided solutions. This structured sequence of content delivery and assessment is detailed in the accompanying figure (Fig. 1), which outlines the architecture of our three-step approach.

The efficacy of the three-step digitization approach was evaluated within the context of Microscopic Anatomy (Histology) lectures at the Medical Faculty of the Ruhr University Bochum. To ensure a robust comparison, we selected two topics of similar difficulty level. One topic was taught using the traditional face-to-face lecture format, while the other was delivered through our digitized format, adhering to the three-step approach outlined in Fig. 1. This methodological choice was critical for isolating the impact of the digitization process on student learning outcomes. Additionally, to minimize potential bias and maintain consistency across the learning experiences, both lecture formats were delivered by the same instructor. In the comparative analysis of our study, both the traditional lecture and the digital module were designed to cover the content within similar time frames, ensuring an equitable basis for comparison. The lecture, delivered in a conventional didactic format, spanned a standard 45-min session, focusing solely on lecture-based instruction without interactive elements. In contrast, the digital module, while also encompassing an equal duration in time, was structured into three distinct phases as part of our three-step approach. This design intended to mitigate the natural decline in concentration over time by interspersing active learning and self-assessment opportunities, thereby potentially reducing the likelihood of distraction.. Following the completion of each lecture scenario, students were invited to participate in a survey designed to assess various aspects of their learning experience.

In this study, we intentionally sequenced the traditional lecture before the digital module to garner fresh impressions and minimize organizational complexities during an active academic term. This decision was aimed at reducing logistical burdens and ensuring that students' evaluations were based on their most immediate lecture experience, thereby aligning with our goal of assessing the digitization approach's effectiveness in a real-world educational setting.We utilized a comprehensive approach to assess the emotional and cognitive aspects of learning within the different learning formats. Drawing from Pekrun et al. [19], we explored a spectrum of emotions that are pertinent to the learning process. To quantify these emotions, we employed a visual analog scale [27], allowing participants to express the intensity of their feelings on a defined range. Specifically, we focused on the constructs of Anxiety and Enjoyment, employing the standardized Achievement Emotions Questionnaire (AEQ) for precise measurement [28]. Beyond emotional responses, our data collection extended to cognitive and preferential aspects of the learning experience. We gathered information on students' concentration during the learning experiences, their learning mode preferences, and the perceived efficiency of each learning setting. This multi-faceted approach enabled us to capture a holistic view of the educational impact of our digitization strategy. To enrich our understanding of the quantitative data, we also collected qualitative feedback on learning environment preferences. This additional layer of data provided insights into the correlations between students' emotional responses, their engagement with the learning material, and their overall satisfaction with the educational formats presented.

Participants eligible for the study were required to be actively enrolled first-semester medical students at Ruhr University Bochum during the data collection period. This requirement was set to target a group of individuals with a relatively uniform educational background. There were no specific criteria regarding age or gender for participation.

The study's participant demographics comprised a total of 318 first-semester medical students from Ruhr University Bochum, with a distribution of 221 females (69.5%) and 97 males (30.5%). The average age of participants was 20.0 years, with a standard deviation of 2.3 years. Female participants had a slightly higher mean age of 20.1 years (SD = 2.2) compared to male participants, who had a mean age of 20,0 years (SD = 2.4). Of the 318 subjects who were offered the opportunity to participate, 462 completed questionnaires were returned, resulting in a total response rate of 231 participants, which led to a representative sample size of around two-thirds of the total cohort.

This research was conducted in compliance with the Declaration of Helsinki and received approval from the Ethics Committee of the Professional School of Education at Ruhr University Bochum (Reference No. EPSE-2023–007, dated 21.09.2023).

In the statistical analysis of our study, we employed a comprehensive set of descriptive statistics to evaluate the distribution and characteristics of positive and negative emotions elicited during both the face-to-face lecture and its digitized counterpart. Specifically, we calculated the median, mean, standard error of the mean (SEM), standard deviation (Std. Dev.), interquartile range (IQR), variance, skewness along with its standard error (Std. E. S.), kurtosis, and the standard error of kurtosis (Std. E. K.) for each emotional tone reported by participants in both learning scenarios.

Our study assessed self-perceived knowledge gain and controlled for self-assessed prior knowledge, rather than directly measuring performance through exams.. Our primary focus was on evaluating learning engagement and the overall learning experience associated with the digitization approach, rather than measuring immediate academic performance through exams. This perspective stems from the understanding that traditional lectures are not designed to assess immediate performance but to foster a learning environment where engagement and conceptual understanding are prioritized. It was achieved by subtracting the pre-course knowledge level from the post-course knowledge level for each individual. To analyze these differences statistically, we conducted paired t-tests, setting the significance level at 0.05. This method allowed us to rigorously evaluate the impact of each teaching method on students' perceived knowledge acquisition.

In addition to the quantitative analysis, we examined qualitative data derived from participants' feedback. We categorized the key arguments presented in the feedback and calculated the frequencies of these categories. This qualitative analysis enabled us to identify prevalent themes and insights regarding the learning experiences in both the traditional and digitized formats, providing a richer understanding of the educational impact of our three-step digitization approach.

## Results

In this section, we present the findings from our study comparing the three-step digitization approach to its traditional face-to-face counterpart in medical education. The analysis encompasses descriptive statistics to outline students' emotional responses, inferential statistics and qualitative analyses, offering a comprehensive view of the impact of our digitization approach versus traditional instruction on both emotional and cognitive outcomes.

Regarding the descriptive statistics of positive emotions—measured on a visual analog scale ranging from minimum 1.00 to maximum 10.00—during the lecture, it can be stated that interest (INT) and motivation (MTV) showed the highest median values at 7.00 and 6.00 respectively. Joy (JOY) and hope (HPE) had median values of 5.00, while pride (PRD) and relaxation (RLX) presented the lowest medians at 4.00 and 3.00. The mean values align closely with the medians, with interest at 7.04 and motivation at 6.13 being the most prominent. Standard deviation values, such as 2.52 for pride (PRD) and 2.35 for hope (HPE), indicate variability in responses. The skewness for interest at -0.49 and kurtosis for pride at -0.99 provide details on the distribution shapes (Table 1).

**Table 1:** Descriptive Statistics of positive emotions during the lecture

	**JOY**	**ENT**	**INT**	**MTV**	**HPE**	**CUR**	**PRD**	**RLX**	**CNT**
Median	5.0000	6.0000	7.0000	6.0000	5.0000	7.0000	4.0000	3.0000	5.0000
Mean	4.8095	5.3853	7.0433	6.1299	4.8528	6.9091	4.3939	3.4805	4.6883
SEM	0.1355	0.1364	0.1197	0.1365	0.1548	0.1203	0.1659	0.1340	0.1425
Std. Dev	2.0596	2.0733	1.8197	2.0748	2.3526	1.8286	2.5222	2.0363	2.1665
IQR	3.0000	3.0000	2.0000	3.0000	3.5000	2.0000	4.0000	3.0000	3.0000
Variance	4.2418	4.2987	3.3112	4.3048	5.5348	3.3439	6.3615	4.1464	4.6937
Skewness	0.2556	-0.0969	-0.4884	-0.2663	0.1256	-0.4369	0.2503	0.6673	0.1201
Std. E. S	0.1601	0.1601	0.1601	0.1601	0.1601	0.1601	0.1601	0.1601	0.1601
Kurtosis	-0.3561	-0.6059	0.2891	-0.2163	-0.7372	0.0622	-0.9981	0.0007	-0.6692
Std. E. K	0.3189	0.3189	0.3189	0.3189	0.3189	0.3189	0.3189	0.3189	0.3189
Minimum	1.0000	1.0000	1.0000	1.0000	1.0000	1.0000	1.0000	1.0000	1.0000
Maximum	10.000	10.000	10.000	10.000	10.000	10.000	10.000	10.000	10.000

*JOY* Refers to joy, *ENT* To enthusiasm, *INT* To interest, *MTV* To motivation, *HPE* To hope, *CUR* To curiosity, *PRD* To pride, *RLX* To relaxation, *CNT* To contentment

Median scores for boredom (BRD), frustration (FRS), and stress (STR) were noted at 4.00, 5.00, and 6.00 respectively, indicating moderate levels of these emotions. The mean scores closely follow, with curiosity (CUR) displaying a high mean of 6.91, frustration showing a mean of 5.22, and stress at 5.84, reflecting notable occurrences of these emotions. Standard deviation values, such as 2.80 for anxiety and 2.73 for worry, demonstrate variability in students' experiences of these negative emotions. The interquartile range (IQR) for most emotions spans from 3.00 to 4.00, suggesting a consistent spread of responses across the cohort. Variance, skewness, and kurtosis values offer additional insights into the distribution of these emotions, with skewness for sadness (SAD) at 1.36 indicating a heavier tail towards higher scores. The maximum scores for all emotions reached 10.00, showing that some students experienced high levels of negative emotions during the lecture (Table 2).

**Table 2:** Descriptive Statistics of negative emotions during the lecture

	**BRD**	**FRS**	**DIS**	**DSP**	**SAD**	**STR**	**DMT**	**ANX**	**WRY**	**SHM**	**CNF**
Median	4.0000	5.0000	4.0000	4.0000	2.0000	6.0000	4.0000	4.0000	5.0000	1.0000	5.0000
Mean	4.2597	5.2208	3.8874	4.3117	2.8615	5.8355	4.1688	4.2641	5.0216	2.2814	5.1169
Std. Error of Mean	0.1521	0.1676	0.1584	0.1713	0.1452	0.1746	0.1650	0.1844	0.1793	0.1244	0.1788
Std. Deviation	2.3110	2.5467	2.4075	2.6040	2.2063	2.6537	2.5077	2.8029	2.7258	1.8913	2.7170
IQR	3.0000	4.0000	4.0000	4.0000	3.0000	4.0000	4.0000	5.0000	4.0000	2.0000	4.0000
Variance	5.3409	6.4858	5.7960	6.7807	4.8677	7.0424	6.2888	7.8561	7.4300	3.5770	7.3819
Skewness	0.4025	0.0548	0.4729	0.3872	1.3558	-0.1886	0.6261	0.5006	0.1265	1.7124	0.1341
Std. Error of Skewness	0.1601	0.1601	0.1601	0.1601	0.1601	0.1601	0.1601	0.1601	0.1601	0.1601	0.1601
Kurtosis	-0.7359	-0.9152	-0.9012	-0.9127	1.3027	-0.9615	-0.4305	-0.9522	-1.1025	2.5508	-1.0590
Std. Error of Kurtosis	0.3189	0.3189	0.3189	0.3189	0.3189	0.3189	0.3189	0.3189	0.3189	0.3189	0.3189
Minimum	1.0000	1.0000	1.0000	1.0000	1.0000	1.0000	1.0000	1.0000	1.0000	1.0000	1.0000
Maximum	10.000	10.000	10.000	10.000	10.000	10.000	10.000	10.000	10.000	10.000	10.000

*BRD* Refers to boredom, *FRS* To frustration, *DIS* To disappointment, *DSP* To desperation, *SAD* To sadness, *STR* To stress, *DMT* To demotivation, *ANX* To anxiety, *WRY* To worry, *SHM* To shame, *CNF* To confusion

Regarding positive emotions during the digital course, median scores indicate a strong presence of interest (INT), motivation (MTV), and curiosity (CUR) at 7.00, alongside contentment (CNT) and relaxation (RLX) at 6.00, reflecting positive engagement with the digital format. The mean scores further support this, with interest at 6.87 and motivation at 6.33 showcasing high levels of engagement. Standard deviation values, such as 2.76 for relaxation (RLX) and 2.71 for pride (PRD), suggest variability in emotional experiences among students. The interquartile range (IQR) for most emotions was between 2.00 and 3.00, indicating consistency in responses. Variance, like 5.41 for joy and 5.57 for enthusiasm, along with skewness and kurtosis values, offer insight into the distribution of these positive emotions, with most showing slight deviations from normal distribution. The maximum scores reached 10.00 for all emotions, indicating that some students experienced high levels of positive emotions in the digital learning environment (Table 3).

**Table 3:** Descriptive Statistics of positive emotions during the digital course

	**JOY**	**ENT**	**INT**	**MTV**	**HPE**	**CUR**	**PRD**	**RLX**	**CNT**
Median	5.0000	6.0000	7.0000	7.0000	5.0000	7.0000	5.0000	6.0000	6.0000
Mean	5.4459	5.6277	6.8745	6.3290	5.6494	6.7403	5.0563	5.6234	6.0390
Std. Error of Mean	0.1531	0.1553	0.1374	0.1538	0.1650	0.1446	0.1786	0.1819	0.1568
Std. Deviation	2.3267	2.3609	2.0886	2.3378	2.5079	2.1972	2.7141	2.7649	2.3835
IQR	3.0000	3.0000	2.0000	3.0000	3.0000	3.0000	4.0000	5.0000	3.0000
Variance	5.4134	5.5738	4.3624	5.4652	6.2896	4.8279	7.3664	7.6445	5.6811
Skewness	0.1111	0.0886	-0.5596	-0.3532	-0.1005	-0.4766	0.1007	-0.0577	-0.1865
Std. Error of Skewness	0.1601	0.1601	0.1601	0.1601	0.1601	0.1601	0.1601	0.1601	0.1601
Kurtosis	-0.6590	-0.7379	-0.2555	-0.6057	-0.7416	-0.3434	-0.9947	-1.1478	-0.5848
Std. Error of Kurtosis	0.3189	0.3189	0.3189	0.3189	0.3189	0.3189	0.3189	0.3189	0.3189
Minimum	1.0000	1.0000	1.0000	1.0000	1.0000	1.0000	1.0000	1.0000	1.0000
Maximum	10.0000	10.0000	10.0000	10.0000	10.0000	10.0000	10.0000	10.0000	10.0000

*JOY* Refers to joy, *ENT* To enthusiasm, *INT* To interest, *MTV* To motivation, *HPE* To hope, *CUR* To curiosity, *PRD* To pride, *RLX* To relaxation, *CNT* To contentment

For negative emotions experienced during the digital course, the median values indicate lower levels of negative emotions, with sadness (SAD) and shame (SHM) at a median of 1.00, suggesting infrequent experiences of these emotions. Frustration (FRS) and confusion (CNF) showed slightly higher medians of 2.00 and 3.00, respectively, indicating a moderate presence. The mean values, such as 3.74 for boredom (BRD) and 3.13 for frustration (FRS), reflect a general trend of lower negative emotional responses in the digital learning environment. Standard deviation and interquartile range (IQR) values demonstrate variability among students' responses, with standard deviation figures like 2.13 for boredom and 2.23 for frustration. Variance, skewness, and kurtosis metrics provide further insight into the distribution, with skewness for sadness at 2.51 indicating a positive skew, and kurtosis for shame at 9.44 suggesting a leptokurtic distribution. The minimum and maximum scores span from 1.00 to 10.00 for all emotions, showing a range of emotional experiences among participants in the digital course (Table 4).

**Table 4:** Descriptive Statistics of negative emotions during the digital course

	**BRD**	**FRS**	**DIS**	**DSP**	**SAD**	**STR**	**DMT**	**ANX**	**WRY**	**SHM**	**CNF**
Median	3.0000	2.0000	2.0000	2.0000	1.0000	3.0000	3.0000	1.0000	2.0000	1.0000	3.0000
Mean	3.7446	3.1299	2.7619	2.4459	1.9913	3.3074	3.4719	2.2944	2.8355	1.7100	3.1645
Std. Error of Mean	0.1402	0.1465	0.1418	0.1266	0.1131	0.1482	0.1510	0.1340	0.1481	0.1028	0.1465
Std. Deviation	2.1305	2.2264	2.1548	1.9236	1.7194	2.2527	2.2951	2.0366	2.2513	1.5626	2.2261
IQR	3.0000	3.0000	3.0000	2.0000	1.0000	4.0000	3.0000	2.0000	2.5000	1.0000	3.0000
Variance	4.5388	4.9570	4.6431	3.7003	2.9564	5.0747	5.2677	4.1478	5.0685	2.4416	4.9554
Skewness	0.6562	1.2491	1.5110	1.7117	2.5138	0.9814	0.9692	2.0745	1.4292	2.8984	1.1842
Std. Error of Skewness	0.1601	0.1601	0.1601	0.1601	0.1601	0.1601	0.1601	0.1601	0.1601	0.1601	0.1601
Kurtosis	-0.1429	1.0026	1.9668	3.0990	7.1805	0.4098	0.3653	4.1126	1.3377	9.4420	0.9966
Std. Error of Kurtosis	0.3189	0.3189	0.3189	0.3189	0.3189	0.3189	0.3189	0.3189	0.3189	0.3189	0.3189
Minimum	1.0000	1.0000	1.0000	1.0000	1.0000	1.0000	1.0000	1.0000	1.0000	1.0000	1.0000
Maximum	10.000	10.000	10.000	10.000	10.000	10.000	10.000	10.000	10.000	10.000	10.000

*BRD* Refers to boredom, *FRS* To frustration, *DIS* To disappointment, *DSP* To desperation, *SAD* To sadness, *STR* To stress, *DMT* To demotivation, *ANX* To anxiety, *WRY* To worry, *SHM* To shame, *CNF* To confusion

**Fig. 2:**
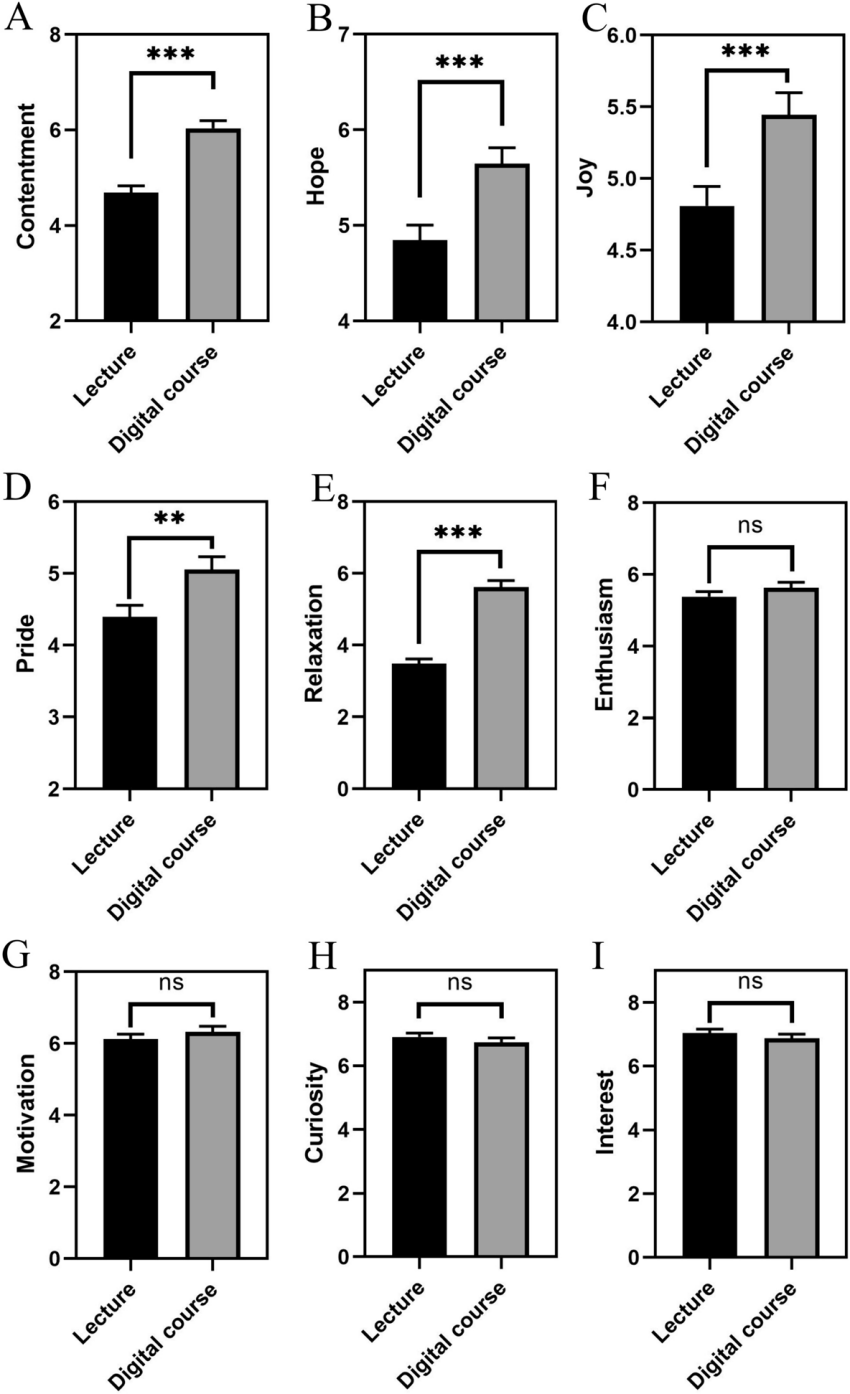
This figure presents bar plots for each measured emotion, contrasting the average levels of positive emotional responses between traditional face-to-face lectures (Lecture) and our three-step digitization approach (Digital course). Each bar represents the mean value of the respective emotion in the traditional and digital settings, with error bars indicating the standard error of the mean (SEM). Significant differences between the two formats are highlighted as; ** denotes *p* < 0.01, *** denotes *p* < 0.001, n.s. denotes not significant

Paired samples t-tests comparing positive emotional responses between a traditional lecture and the three-step digitization approach revealed significant findings. Joy (JOY), hope (HPE), pride (PRD), relaxation (RLX), and contentment (CNT) all showed significant increases in the digital setting, with t-values of -3.579 (*p* < 0.001) for joy, -3.958 (*p* < 0.001) for hope, -3.209 (*p* = 0.002) for pride, -9.800 (*p* < 0.001) for relaxation, and -6.228 (*p* < 0.001) for contentment. These results suggest a notable enhancement of these emotions through digital learning. Conversely, enthusiasm (ENT), interest (INT), and motivation (MTV) displayed no significant differences between the formats, with p-values of 0.174, 0.283, and 0.287, respectively (Fig. 2).

The analysis of negative emotions via paired samples t-tests between the traditional lecture and the digitized lecture showed a significant reduction in negative emotions in the digital format. Specifically, feelings of boredom (BRD) were lower in the digital setting, t(230) = 2.575, *p* = 0.011. More pronounced reductions were observed for frustration (FRS), t(230) = 10.339, *p* < 0.001; disappointment (DIS), t(230) = 5.870, *p* < 0.001; desperation (DSP), t(230) = 9.991, *p* < 0.001; sadness (SAD), t(230) = 5.695, *p* < 0.001; stress (STR), t(230) = 12.999, *p* < 0.001; demotivation (DMT), t(230) = 3.458, *p* < 0.001; anxiety (ANX), t(230) = 9.992, *p* < 0.001; worry (WRY), t(230) = 11.127, *p* < 0.001; shame (SHM), t(230) = 4.041, *p* < 0.001; and confusion (CNF), t(230) = 9.486, *p* < 0.001.

**Fig. 3:**
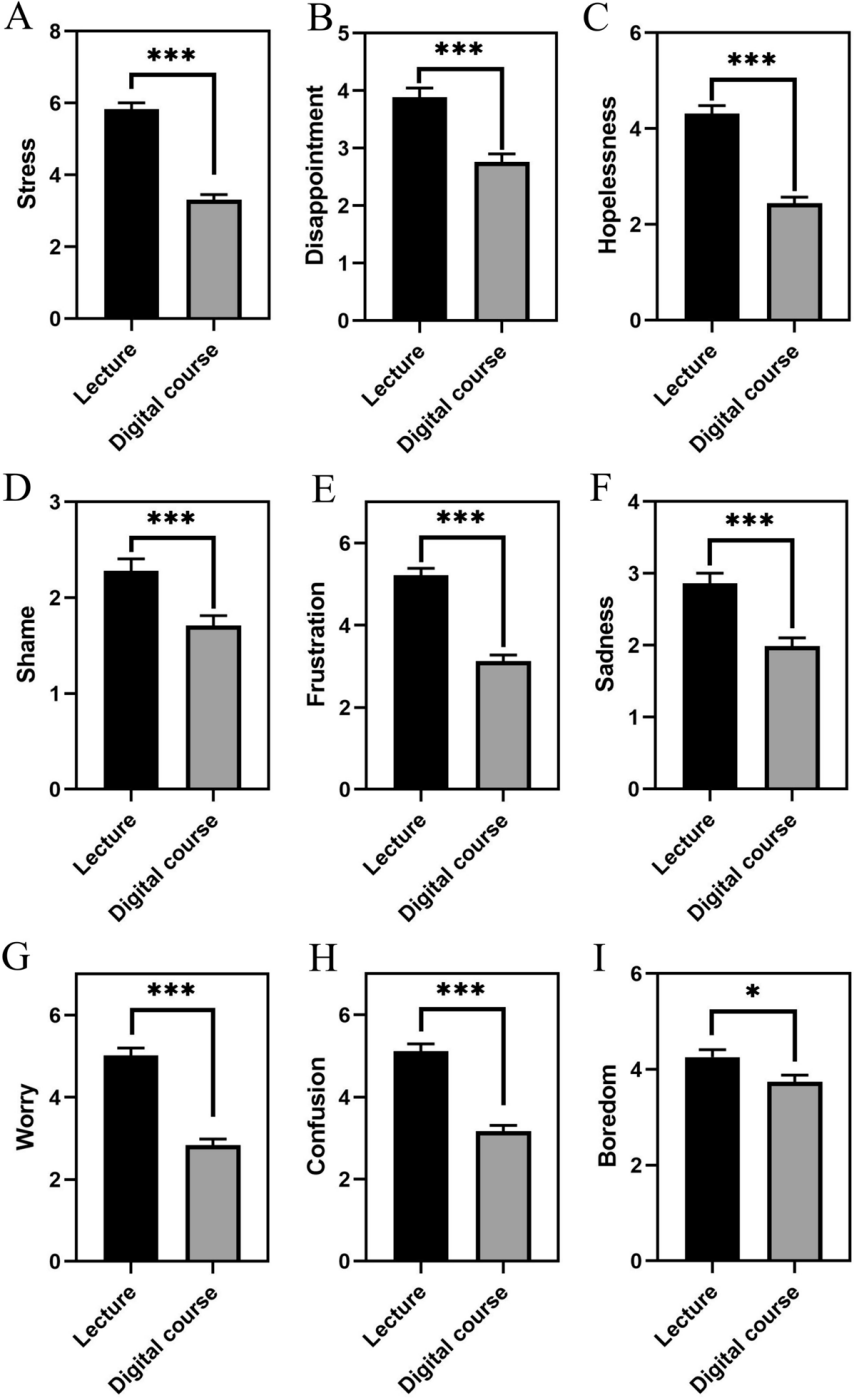
This figure presents bar plots for each measured emotion, contrasting the average levels of negative emotional responses between traditional face-to-face lectures (Lecture) and our three-step digitization approach (Digital course). Each bar represents the mean value of the respective emotion in the traditional and digital settings, with error bars indicating the standard error of the mean (SEM). Significant differences between the two formats are highlighted as; * denotes *p* < 0.05 and *** denotes *p* < 0.001

These results indicate that participants experienced significantly fewer negative emotions during the digital course compared to the face-to-face lecture (Fig. 3).

**Fig. 4:**
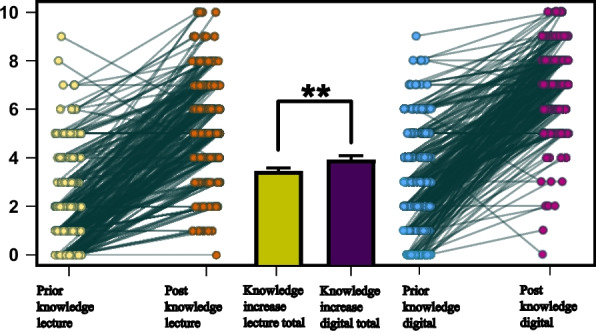
The figure shows scatterplots depicting the subjects' prior knowledge for both the lecture (light yellow) and the digitized lecture (light blue) as well as the perceived knowledge after the lecture (orange) and after the digitized lecture (light purple). The respective difference is shown in the center of the figure, where the increase in knowledge from the lecture (ochre yellow) and from the digitized lecture (dark purple) are depicted. Significant differences between the two formats are highlighted as; ** denotes *p* < 0.01

The comparison of knowledge gain between traditional face-to-face lectures and digitized lectures was assessed through a paired samples t-test, accounting for prior knowledge in both scenarios. The analysis revealed that knowledge gain in the digitized format was significantly higher than in the traditional lecture setting. Specifically, the t-test showed a statistically significant difference in knowledge acquisition favoring the digitized approach, with a t-value of -2.795 (df = 230, *p* = 0.006) (Fig. 4). This indicates that students experienced a greater enhancement of their understanding and retention of the material when engaged with the content through the digitized learning format.

**Fig. 5:**
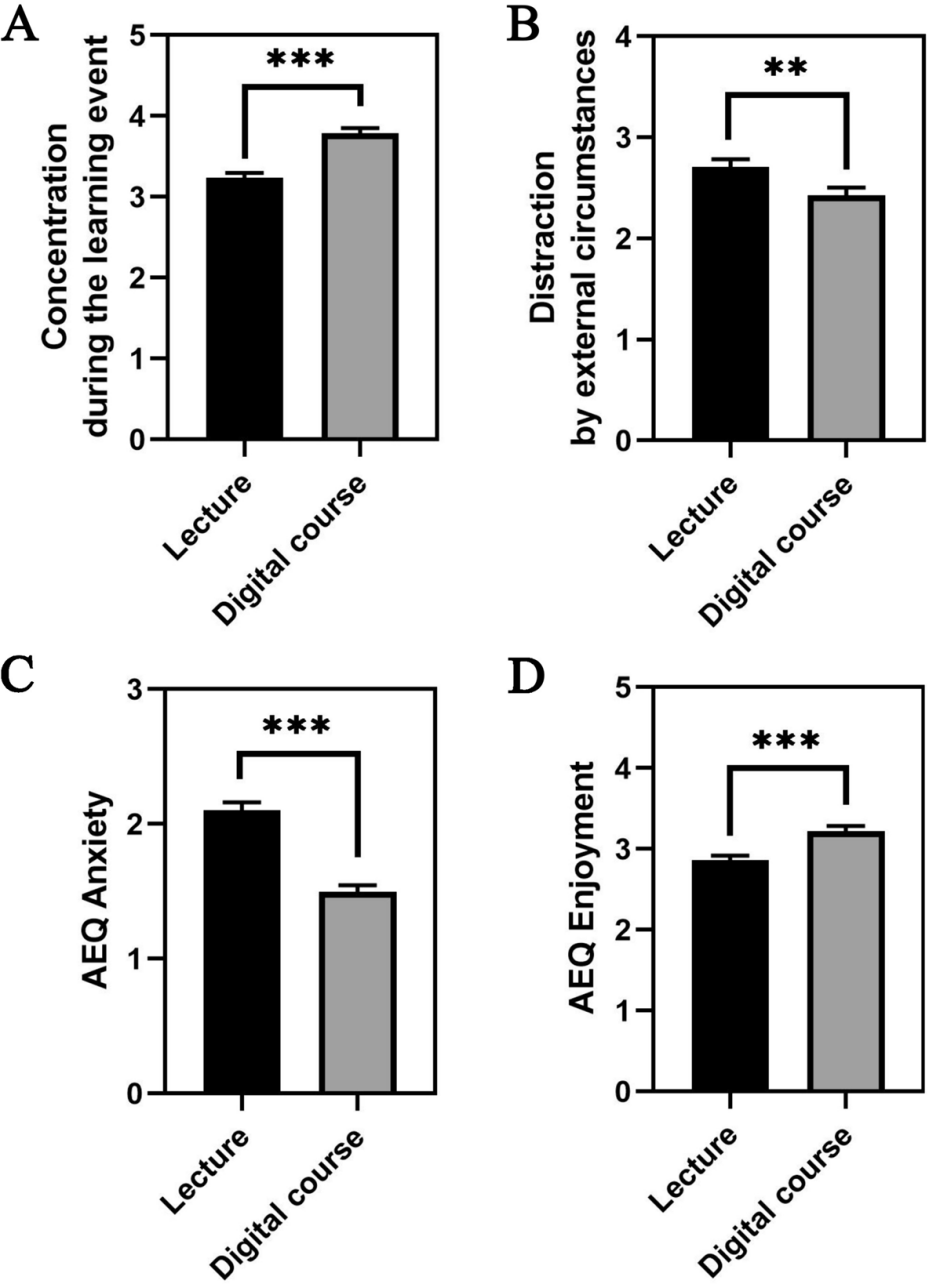
Chart **A** illustrates concentration levels, indicating enhanced focus in digital settings. Chart **B** assesses distractions from external factors, with digital learning showing reduced interference. Charts **C** and **D**, using the AEQ, reveal lower anxiety and higher enjoyment in digital formats, respectively. Each chart presents mean values with error bars indicating SEM. Significant differences between the two formats are highlighted as; ** denotes *p* < 0.01 and *** denotes *p* < 0.001

Analysis for perceived levels of concentration and distraction offered insightful contrasts between the traditional lecture hall setting and the online learning environment. Notably, students reported significantly higher levels of concentration when participating in online learning, as indicated by a t-value of -5.801 (df = 230, *p* < 0.001) (Fig. 5A). Substantiating this finding, the analysis regarding perceived distractions revealed that students experienced a higher level of distraction in the face-to-face lecture setting compared to the online environment. The statistical outcome, with a t-value of 2.848 (df = 230, *p* = 0.005) (Fig. 5B), supports the notion that the traditional classroom setting may present more elements that divert attention away from the learning material.

Building on the previous findings that online learning environments potentially can enhance concentration while reducing distractions, the Achievement Emotions Questionnaire (AEQ) results further illuminate the emotional benefits of our digitization approach. Specifically, the AEQ results revealed a significant decrease in anxiety levels in the online learning environment, as shown by a t-value of 9.446 (df = 230, *p* < 0.001) (Fig. 5C). In parallel, enjoyment levels significantly increased, as shown by a t-value of -4.717 (df = 230, *p* < 0.001), indicating heightened enjoyment in our online setting (Fig. 5D).

**Fig. 6:**
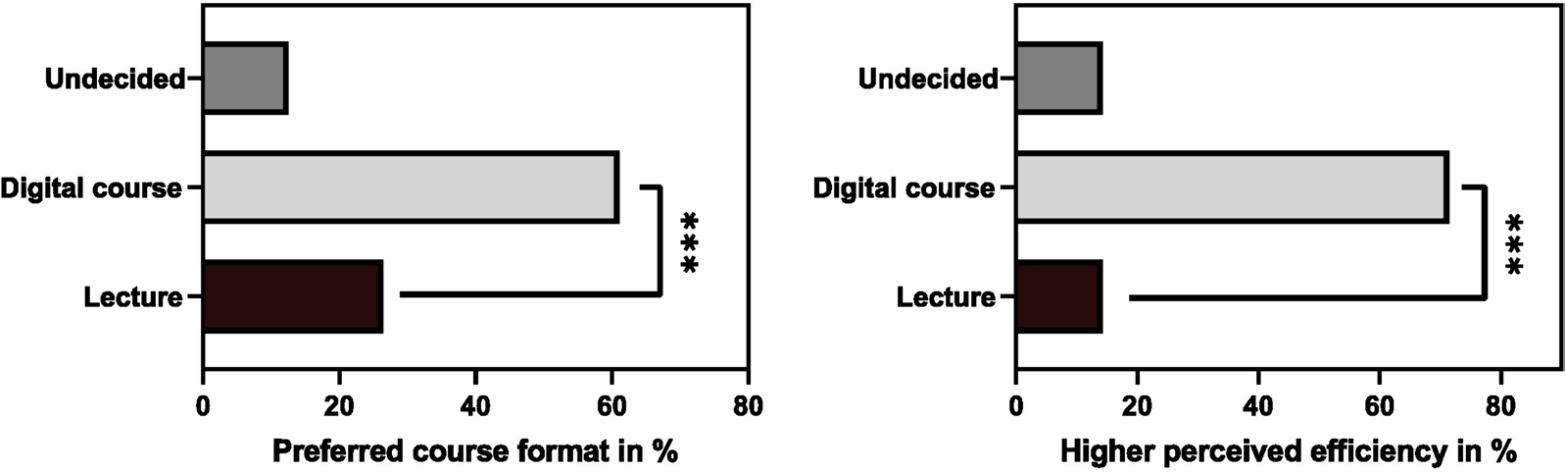
**A** shows student preferences for traditional lectures, our digital course, or undecided. **A** significant majority prefer the digital course format. **B** illustrates perceptions of learning efficiency between traditional lectures and digital courses, with an option for undecided. The majority view the digital format as more efficient, with data presented in percentages. *** denotes *p* < 0.001

In assessing student preferences and perceptions regarding course format, our findings reveal a distinct inclination towards the digital learning environment introduced by our three-step digitization approach. According to the collected data, a significant majority of students (61.0%) expressed a preference for the digital course format over the traditional lecture (26.4%), with a small portion remaining undecided (12.6%) (Fig. 6A). Further analysis aimed to discern whether this preference was merely due to convenience or attributed to perceived educational efficiency. The results unequivocally showed that students regard the digital learning setting as more efficient, with a substantial 71.4% endorsing the digital course for its efficacy, compared to 14.3% favoring the traditional lecture format, and an equal percentage (14.3%) remains undecided (Fig. 6B).

Our qualitative analysis of student feedback on the digitized lecture format revealed insightful perspectives on its advantages and disadvantages. Positive feedback emphasized the digital format's flexibility, with 104 mentions (45.0%) of flexible time management as a significant benefit, allowing students to tailor their learning schedules to personal needs. The extensive and versatile learning experience was highlighted by 99 (42.9%) participants, appreciating the diversity in learning materials and approaches. Direct feedback on learning progress was noted 43 (18.6%) times as a key advantage, fostering a sense of immediate understanding and improvement. Improved concentration and stress reduction were also mentioned, with 21 (9.1%) and 31 (13.4%) mentions respectively, indicating an environment conducive to focused learning and lower anxiety levels. Conversely, negative feedback centered on aspects unique to traditional lectures. Twenty-eight (12.1%) participants felt a better understanding of material during in-person lectures, pointing to potential limitations in digital content delivery. The absence of a social component in digital settings was a concern for 16 (6.9%) respondents, suggesting a missed opportunity for peer interaction and support. A preference for auditory learning and the benefit of fixed lecture schedules in providing daily structure were mentioned by 11 (4.8%) and 6 (2.6%) participants, respectively, highlighting personal learning preferences and organizational benefits associated with face-to-face lectures. This qualitative feedback underscores the complex balance between the digital format's convenience and effectiveness versus the perceived depth of understanding and social interaction offered by traditional lectures.

## Discussion

The investigation into our three-step digitization approach for medical education reveals its substantial impact on enhancing learning experiences, marking a significant stride towards meeting the exigencies of modern educational frameworks. Designed to transform traditional lectures into dynamic digital formats, this approach not only caters to the current demands regarding current challenges in the field of Medical Education but also confronts the prevailing challenges within academic digital environments.

Elevated expressions of positive emotions such as enjoyment, contentment, hope, and pride in our digitized lecture format are corroborated by existing research, indicating a substantial impact of these emotions on learning outcomes. Positive emotions enhance the link between cognitive/motivational variables and academic achievement, suggesting that higher levels of enjoyment and pride can make self-regulation strategies more effective in improving grades [29]. The importance of positive emotions in promoting academic success is further emphasized, with activity-related emotions like enjoyment identified as critical for academic performance [30]. The interplay between emotions and cognitive processes, including attention, memory, and motivation, underscores the crucial role of emotional well-being for optimal learning [31]. Moreover, learning-related emotions and metacognitive strategies are shown to mediate the relationship between self-efficacy and academic performance, highlighting the intertwined nature of emotional states and learning strategies [32]. The interrelation between certain emotional characteristics in academic learning environments and the generally perceived basic tone of certain affective characteristics, further supports the significance of fostering these emotions within educational settings [33].

Transitioning to the implications of negative emotions, the Control-Value Theory (CVT) provides a framework for their impact, illustrating how perceptions of control and value attached to learning activities influence emotional experiences: Positive emotions are associated with high control and value, whereas negative emotions emerge from perceived low control or value [34, 35]. This highlights the necessity of addressing both positive and negative emotions to fully understand and enhance the learning environment, paving the way for strategies aimed at minimizing negative emotional impacts on academic achievement.

In academic contexts, negative emotions like stress, disappointment, and frustration are generally seen as barriers to learning, correlating with lower academic achievement and a surface approach to learning [36]. Similarly, Pekrun et al. [37] highlight a negative cycle where such emotions adversely impact performance. Our findings indicate that the three-step digitization approach significantly reduces these negative emotions, aligning with literature that underscores the importance of minimizing such emotional experiences to enhance learning outcomes. While there is some discussion in the literature about the potential constructive roles of negative emotions under certain conditions [18, 38], the primary focus of our study — and the broader consensus in educational research — is on the detrimental impact of these emotions on learning processes. By effectively reducing negative emotions, our digitization approach contributes to creating a more positive and conducive learning environment, which is crucial for academic success.

Our research on knowledge gain perceived within our three-step digitization approach setting aligns with findings that digital technologies can enhance educational outcomes. It's demonstrated that when digital tools facilitate constructive and interactive engagement, learning outcomes are positively impacted [39]. Blended learning strategies, which combine digital and traditional methods, have shown to be effective in health education, supporting the integration of digital technologies into teaching methodologies [40]. Additionally, studies suggest that digital learning designs, such as blended and distance learning, contribute positively to student learning outcomes, emphasizing the role of digital formats in education [41]. The emphasis on integrating technology in education, aligning with pedagogical principles, reflects a broader educational trend towards enhancing learning experiences and outcomes through digital means. This approach is advocated to transform education for future health professionals, resonating with our findings that a structured digitization approach can enrich the learning process [42]. However, considering the blended educational backgrounds of our participants, who had previous face-to-face lecture experiences, it's plausible that the observed benefits of the digital format may also derive from the cumulative advantages of a blended approach. This notion underscores the complexity of comparing digital and traditional learning modalities, highlighting the importance of considering the interplay between different educational experiences in assessing their effectiveness.

Further, our data reveal that students experienced less distraction and higher concentration during the learning process. This aligns with previous research arguing that auditory distractions, especially language, can significantly impair recall by drawing cognitive resources away from task-salient information [43]. Our digital environment likely minimized such distractions by providing a focused, coherent stream of input, enhancing students' ability to concentrate and recall lecture content. Furthermore, the research by Shernoff et al. [44] on the impact of seating location on student engagement and attention in traditional lecture settings underscores the importance of environmental factors in learning concentration, emphasizing the temporary advantage of a potentially calm learning atmosphere in a remote learning environment.

Our three-step digitization approach presents a transformative solution in education, characterized by its ease of use and comprehensive benefits for enhancing the learning experience. By reducing negative emotions and amplifying positive ones, it creates an optimal learning environment that fosters student well-being and engagement. The approach effectively minimizes distractions and enhances concentration, leading to improved efficiency and academic performance. It offers students unparalleled flexibility in managing their learning schedules, coupled with a versatile and enriched learning experience. The inclusion of direct feedback mechanisms further supports immediate learning adjustments and deeper comprehension. Altogether, this approach embodies a forward-thinking response to modern educational demands, providing a structured yet flexible framework that adapts to the varied needs of learners and maximizes their potential for success.

While our study highlights the benefits of the three-step digitization approach, it's important to consider its limitations alongside proposing relevant future research directions. We recognize the potential for a sequence effect due to the fixed order of the learning formats. This design choice was made to ensure fresh impressions and ease the logistical challenges of executing a crossover design within an ongoing semester. A crossover design, although ideal for mitigating sequence bias, would necessitate multiple iterations of the same lecture and could potentially lead to inter-cohort bias through communication among students. Future studies could explore alternative methodologies that both mitigate sequence effects and address the practical challenges identified in implementing crossover designs in educational research. Our study's design thus opens avenues for further investigation, highlighting the need for innovative solutions to balance methodological rigor with operational feasibility in educational settings. While our study provides valuable insights into the efficacy of the three-step digitization approach in medical education, we recognize the limitations in directly generalizing these findings to all educational contexts. Future research should explore the adaptability of this approach across various disciplines and student populations. Future research should explore its applicability and effectiveness in a broader range of academic fields, contributing to a holistic understanding of how digital learning can complement traditional teaching methods. Further, our assessment strategy was designed to align with our primary interest in evaluating the digitization approach's effectiveness from the learners' perspective. Future studies are encouraged to incorporate performance-based assessments to further elucidate the digitization approach's impact on student learning outcomes across multiple learning sessions. The potential for long-term enhancements in learning outcomes through our digitization approach merits further investigation. We advocate for longitudinal studies to assess how such digital strategies influence knowledge retention and application over time. This exploration is crucial not with the aim of replacing face-to-face lectures entirely but rather to develop a blended learning model where digitization serves to augment and diversify the educational experience. Such a model would leverage the strengths of both digital and traditional formats, providing flexibility, enhanced engagement, and potentially greater learning outcomes. We invite the academic community to further evaluate this approach across different disciplines and learning objectives, viewing it as an opportunity to innovate and enhance educational strategies within a complementary framework that retains the invaluable elements of in-person teaching.

## Conclusion

In conclusion, our three-step digitization approach was crafted to offer educators an accessible and straightforward method for transforming traditional lectures into engaging digital content, even without extensive didactic expertise. The approach has been positively evaluated, showcasing its potential to enhance learning experiences through reduced distractions, increased concentration, and improved emotional well-being among students. While our findings within the realm of microscopic anatomy are promising, the true versatility and applicability of this approach across different disciplines and educational scenarios remain to be fully explored. Future research is essential to uncover the breadth of its effectiveness and to identify other areas where it can be successfully applied. This initiative paves the way for a broader adoption of blended learning models, combining the best of digital and traditional teaching methods to enrich the educational landscape.

### Supplementary Information

Supplementary Material 1.

## Data Availability

The datasets generated and analyzed during the current study are available from the corresponding author on reasonable request.
