# Supplementary figures and images for: Adapting to new challenges in medical education: a three-step digitization approach for blended learning
Source: BMC Med Educ. 2024 May 28;24:585. doi: 10.1186/s12909-024-05503-1 (PMC11134748; doi:10.1186/s12909-024-05503-1)

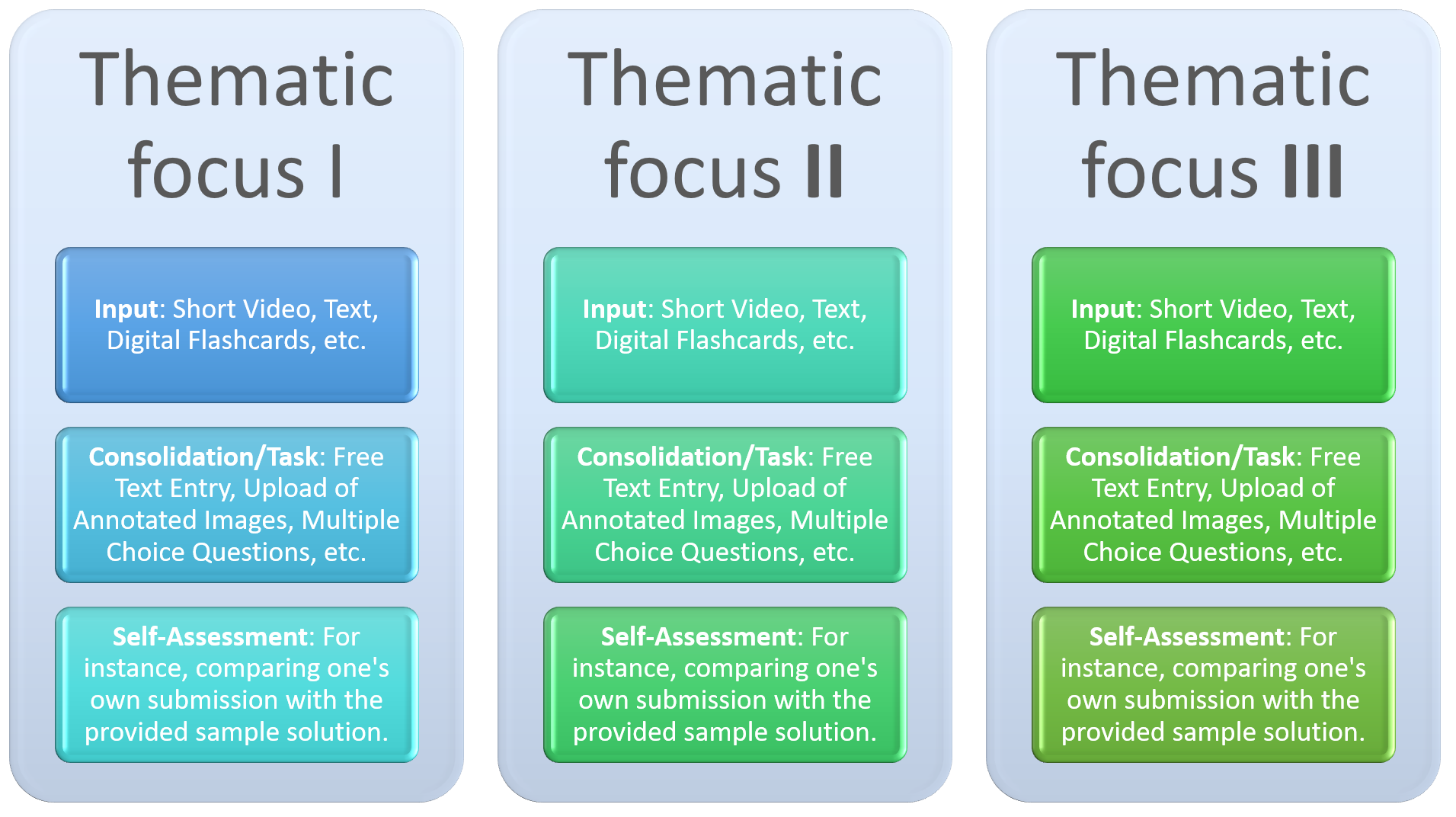

Supplement: Supplementary file 1 — Supplementary Material 1. [file 12909_2024_5503_MOESM1_ESM.png]
